# Understanding health behaviour in pregnancy and infant feeding intentions in low-income women from the UK through qualitative visual methods and application to the COM-B (Capability, Opportunity, Motivation-Behaviour) model

**DOI:** 10.1186/s12884-018-2156-8

**Published:** 2019-02-12

**Authors:** Aimee Grant, Melanie Morgan, Dawn Mannay, Dunla Gallagher

**Affiliations:** 1 Centre for Trials Research, Cardiff University Neuadd Meirionnydd, Heath Park, Cardiff, CF14 4YS UK Wales; 2 School of Social Sciences, Cardiff University, Glamorgan Building, King Edward VII Avenue, Cardiff, CF10 3WT UK; 3 Centre for Public Health, Institute of Clinical Sciences, Queens University Belfast, Belfast, UK Ireland

**Keywords:** Pregnancy, Antenatal, Smoking, Alcohol, Infant feeding, Breastfeeding, Qualitative, Visual methods, Creative methods, COM-B model

## Abstract

**Background:**

Health behaviours during pregnancy and the early years of life have been proven to affect long term health, resulting in investment in interventions. However, interventions often have low levels of completion and limited effectiveness. Consequently, it is increasingly important for interventions to be based on both behaviour change theories and techniques, and the accounts of pregnant women. This study engaged with pregnant women from deprived communities, to understand their subjective experiences of health in pregnancy.

**Methods:**

The study adopted a women-centred ethos and recruited a purposive sample of ten pregnant women, who lived in deprived areas and were on low incomes. Participants engaged with three creative techniques of visual data production (timelines, collaging and dyad sandboxing), followed by elicitation interviews. One participant only engaged in the initial activity and interview, resulting in a total of 28 elicitation interviews. This in-depth qualitative approach was designed to enable a nuanced account of the participants’ thoughts, everyday experiences and social relationships. Data were deductively coded for alcohol, smoking and infant feeding and then mapped to the COM-B model (Capability, Opportunity, Motivation – Behaviour).

**Results:**

Five participants had experience of smoking during pregnancy, four had consumed alcohol during pregnancy, and all participants, except one who had exclusively formula fed her child, disclosed a range of infant feeding experiences and intentions for their current pregnancies. Considerable variation was identified between the drivers of behaviour around infant feeding and that related to abstinence from tobacco and alcohol during pregnancy. Overall, knowledge and confidence (psychological capability), the role of partners (social opportunity) and support from services to overcome physical challenges (environmental opportunity) were reported to impact on (reflective) motivation, and thus women’s behaviour. The role of the public in creating and reinforcing stigma (social opportunity) was also noted in relation to all three behaviours.

**Conclusions:**

When designing new interventions to improve maternal health behaviours it is important to consider the accounts of pregnant women. Acknowledging pregnant women’s subjective experiences and the challenges they face in negotiating acceptable forms of motherhood, can contribute to informed policy and practice, which can engage rather than isolate potential user groups.

**Electronic supplementary material:**

The online version of this article (10.1186/s12884-018-2156-8) contains supplementary material, which is available to authorized users.

## Background

The negative impact of health behaviours during pregnancy, including smoking [1] and drinking alcohol [2], on outcomes in infancy and beyond has been clearly established. Within Western countries, this has resulted in guidance on maternal behaviours which should be avoided. For example, in the United Kingdom (UK), abstinence from smoking and medicinal nicotine containing products (such as Nicotine Replacement Therapy) is recommended throughout pregnancy [3]. In January 2016, UK guidance changed to recommend complete abstinence from alcohol during the pre-conception period and throughout pregnancy [4]. It should be noted this change to guidance has been contentious in public health circles, where a lack of robust epidemiological evidence is highlighted [5]. However, qualitative research with women in Australia, found that overall women accept the guidance to be abstinent in principle, even if they do not follow it themselves [6]. In regards to infant feeding, the UK National Health Service advocates World Health Organization guidance in relation to infant feeding, recommending exclusive breastfeeding for the first six months, and breastfeeding alongside the introduction of solid food until at least two years of age [7, 8].

In many Western countries, investment is being made in public health interventions intended to support women to meet this public health guidance. The majority of interventions focus on changing the behaviour of the individual woman. Arguably, this emphasis can assume the superiority of the foetus over the mothers’ health [9], and neglect the social context of the health behaviour in women’s lives [10]. Pregnant women have reported that interventions may be rushed, judgemental and didactic [11] and can reinforce the behaviour they seek to prevent through shaming participants [12].

Midwives involved in the delivery of these interventions have highlighted a lack of time, training and resources to facilitate these roles [13]. It is therefore unsurprising that many interventions targeted at women from the most deprived areas, or who engage in behaviours that are detrimental to healthy pregnancies, often have low uptake and high dropout [14]. However, even where interventions have engaged women, they have not necessarily delivered benefits in terms of health behaviours in pregnancy, such as reduced smoking [15]. The use of financial incentives has been found to be cost effective in relation to maternal smoking cessation [16, 17] and feasible to deliver in relation to infant feeding [18]. However, this approach is not currently accepted by policymakers [19], health professionals [20] or the public [21], so is unlikely to be widely adopted.

An alternative way to improve health behaviour in pregnancy and the early years may be to examine the social-environmental contexts of behaviours, in order to devise supportive interventions to enable health promotion at a societal level [22]. For example, a wide range of adaptations are recommended to create a society that is breastfeeding-friendly [23]. One approach to theorising behaviour in this way is the COM-B model [24]. The COM-B model proposes that ‘Behaviour’ can be understood as a result of Capability, Opportunity, and Motivation (see Table 1). These three factors interact to produce behaviour, and may explain the differences in pregnancy related health behaviour among socio-economic groups, who have different Capability and Opportunity to change behaviour, regardless of Motivation. Arguably, a lack of consideration of Capability, Opportunity and Motivation within an individuals’ environment, limits the potential impact of interventions, as they do not address wide ranging influences on behaviour.

**Table 1:** COM-B domains (based on Michie et al., 2014)

	Domain	Examples
Capability	Physical	Skills, strength, stamina
	Psychological	Knowledge, confidence, memory
Opportunity	Social	Social norms, interpersonal influences
	Environmental	Resources, physical environment
Motivation	Automatic	Impulses, desires, addiction
	Reflective	Beliefs, intentions

As many interventions targeted towards deprived women in pregnancy are either not possible to deliver at a sufficient dose, or are not effective, it is important to generate a solid understanding of the baseline behavioural system in pregnant women, ahead of attempting to design interventions. Service users’ views have been incorporated into the COM-B model in a range of studies including a survey of Australian women regarding diet in pregnancy [25], infant feeding [26] Aboriginal women in relation to smoking cessation [27], and Kuwaiti women in relation to oral care in pregnancy [28]. Furthermore, this research has been used to develop new interventions. For example, low income UK women were engaged in designing a new breastfeeding peer support intervention using the COM-B model [29] and this intervention was subsequently found to be acceptable to women and health professionals [30]. To date, however, a broader understanding of multiple health behaviours in pregnancy among low income women has not been applied to the COM-B model. This is of particular relevance in maternal health, as due to the time-critical nature of healthy pregnancy interventions, multiple public health interventions need to be offered as a matter of urgency [31]. These diverse interventions are often delivered by midwifery staff as part of usual maternity care pathways [13].

## Methods

Our research was situated in an interpretivist paradigm as it was primarily interested in the subjective perspectives, meaning making and understandings of the participants. It aimed to: (i) use creative methods with pregnant women living in deprived areas on a low income in the UK to facilitate discussion of experiences and thoughts in relation to health behaviours and pregnancy; (ii) to map these findings to the COM-B Model; and (iii) to report this research in line with COREQ guidelines (Additional file 1) [32]. The research design was directly informed by a workshop with 12 women who had children aged under two years who attended a pre-existing mother and baby group in a deprived area of south Wales, UK. This form of consultation prior to conceptualising the research design sought to bridge the divide between academic research and practitioner and service user needs, and maximise the impact and influence of research study.

### Study design and sample

The research aimed to centre and value the participants’ subjective accounts, positioning them as ‘experts by experience’ [33]. This was important as women in low income areas are often marginalised and have little voice in the policies that impact their lives. The approach was interested in both current experiences and the ways in which these were impacted by participants’ wider biographies. Additionally, the design moved away from the research led ‘question and answer’ format by introducing creative activities, which enabled participants to lead and direct the discussions around the data that they produced [34–36]..

To ensure that the research was seen as impartial, and not related to the health service, a purposive sample of participants were recruited externally from the health service. We recruited participants from the research team’s social networks using face-to-face discussions with family, friends and acquaintances (*n* = 2), flyers and personal recommendations from staff at well attended mother and baby groups (*n* = 6), and through local social media groups aimed at mothers (n = 2). We also attempted to recruit participants using flyers in local communities that were part of the Flying Start programme and where the researchers had a local connection, but did not recruit any participants using this approach. Due to this multi-faceted approach it is not possible to give a precise number of those who chose not to engage in the study. In recruitment materials, the study was titled “Health and Wellbeing in Pregnancy”, to reduce the stigma associated with risky health behaviours in pregnancy. The recruitment materials emphasised that we wanted women to tell their story through the use of creative tasks and interviews taking around four hours of their time, and that we would be able to thank them for their time through the use of shopping vouchers (£25 per phase, up to a maximum of £50). We recruited 10 women who were less than 30 weeks pregnant at the time of their first interview. All women were pregnant, resident in areas of the highest quintile of deprivation according to the Welsh Index of Multiple Deprivation [37], and were claiming means tested (welfare) benefits, as per the study’s inclusion criteria. All participants were involved in phase one, nine of the original 10 participants took part in the second data collection period. The 10th participant was lost to follow up.

### Researchers and positionality

Three female researchers were involved in the fieldwork. Melanie a post-doctoral research assistant who has two adult children, Dawn, a senior lecturer who has adult children and young grandchildren and Dunla a research assistant and doctoral student who was pregnant during data production. Aimee, an experienced qualitative researcher, who does not have children, provided weekly support to Melanie and Dunla. Dawn provided specialist methodological support on a monthly basis. Researcher positionality [38] and personality [39] impact on the data produced. For example, one participant who did not disclose smoking during pregnancy to Dunla, subsequently disclosed this to Dawn. This illustrates the ways in which the commonality of motherhood was further complicated by other characteristics, such as age, class, biography, and national and local identity. All researchers kept field diaries in order to aid reflexivity.

### Ethics, consent, permissions and permission to publish

All participants were asked to provide written informed consent to take part in the research. Participants chose whether they gave consent for their interviews to be audio recorded and/or for anonymised quotations to be used in reporting findings; all participants agreed to both of these voluntary permissions. All participants were allocated a pseudonym. The research was granted ethical approval by Cardiff University School of Medicine Research Ethics Committee.

### Data production

**Fig. 1:**
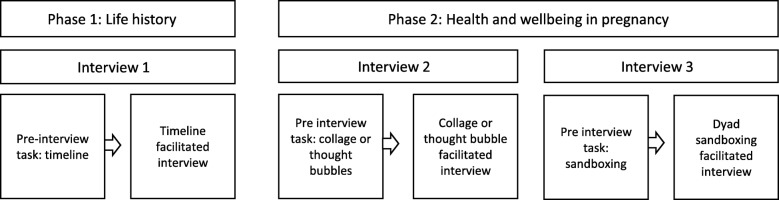
Data production process

Data were produced through a pre-interview task followed by an elicitation interview, and this process was repeated three times using different creative tasks (see Fig. 1). In the instructions that accompanied the pre-interview packs, which were sent by post, and in all telephone/text message correspondence with the research team participants were reminded of the study’s aim and funding and given a choice of how to engage with the task, and they could select which information to include and exclude. The elicitation interviews were conducted during two phases, with interview one occurring in phase one, and interviews two and three occurring on the same day in phase two. Phases one and two were, approximately one month apart at the convenience of the interviewee. All data were produced between March and August 2016. All interviews occurred in participants’ homes. Non-participants, including partners, children and occasionally other family members, were sometimes present.

During the first phase, participants were asked to create a timeline of their life prior to the interview [40], and were sent a resource pack including a timeline template, a range of alternative paper to use, and coloured stickers and pens to represent emotions [41]. Instructions asked participants to: “write a brief timeline of key events in your life”. This method enabled the participants to reflect on their lives ahead of the interview, and to direct their life history interview through reference to their timeline, leading the direction and pace of the interview. The purpose of a life history interview was to situate each woman’s experiences of being pregnant and of risky health behaviours in the context of their wider biographies. All 10 participants took part in this first phase of data production.

During phase two, participants undertook a further two interviews. Participants were sent a second pre-interview pack, which included (i) materials for producing a collage - a range of coloured papers, stickers and glue and (ii) a template which contained a picture of a pregnant woman’s torso surrounded by thought bubbles. In these pre-tasks, participants were asked to consider: “how being pregnant impacts your everyday life”. The option of a primarily visual or primarily written pre-task, enabled participants to select an activity which best suited their preferences. This pre-task was designed to encourage participants to focus on the lived experience of their current (and any previous) pregnancies.

Following the second elicitation interview, the researcher and participant engaged in a third creative activity, dyad sandboxing [42]. The researcher and participant each used a sand-tray and a range of figures to metaphorically describe: “what pregnancy is like on a day to day basis”. The sandboxing activity enabled metaphoric communication through a range of 3D figures and objects to reflect further on the themes arising in interview two. This final elicitation interview took a dyad approach, involving the participant and researcher, for two reasons. Firstly, on a practical level, participants would be less likely to feel self-conscious in creating a sand-scene if the researcher was similarly occupied. Secondly, the shared nature of the activity both enabled a more equal encounter, and allowed conversations to develop around the experiences of both the researchers and the participants [39].

The researcher and participant generally sat separately and quietly spent around 10 min creating a sand-scene. This part of phase two was not audio recorded. When the sand-scene was complete, the researcher and participant sat together for the third and final audio-recorded elicitation interview. First the participant described their experiences of pregnancy through their sand-scene, and then the researcher used their own sand-scene in the same way. Areas of similarity and difference were discussed. This joint engagement led to new conversations and further insights around health behaviours, which would not have arisen without the researchers openly sharing their own experiences of pregnancy.

Overall, participants responded well to requests to produce visual data. Only two of the 28 interviews were conducted without a participant created visual artefact (see Table 2). These two participants highlighted that the task was daunting, but further guidance and reassurance enabled them to produce visual data in both interviews within phase 2. The use of multiple creative methods across the three interviews, each tailored to the specific focus of the interview, allowed participants to reflect on their experiences. Participants found it interesting to reflect on their lives, and although some discussed distressing memories, they still found the process productive. This suggests that the study’s design worked well to both engage participants and share their experiences in a supportive environment.

**Table 2:** Data produced and engagement with participatory visual method tasks

Participant	Phase 1		Phase 2				Total word count
	1st Pre-Interview Timeline Task	Phase 1 Word count	2nd Pre-Interview Thought Bubbles Task	2nd Pre-Interview Collage Task	3rd Pre-interview Sandboxing task	Phase 2 Word count	
Anna	*✓*	21,462	*✓*	*✓*	*✓*	15,294	36,756
Becky	*✓*	2836*	*✓*	x	*✓*	12,584	15,420
Cat	*✓*	8043	x	x	*✓*	12,292	20,335
Donna	*✓*	17,036	*✓*	*✓*	*✓*	17,792	34,828
Ellie	*✓*	6680	n/a	n/a	n/a	n/a	6680
Fiona	*✓*	4787	x	*✓*	*✓*	7824	12,611
Gaby	*✓*	9714	x	*✓*	*✓*	11,494	21,208
Hayley	*✓*	9116	*✓*	x	*✓*	12,253	21,369
Imogen	x	6532	*✓*	x	*✓*	10,237	16,769
Jess	x	9339	*✓*	x	*✓*	11,130	20,469
Total	8	95,545	6	4	9	110,900	206,445

*Audio recording error

### Data analysis

Interviews were audio recorded and fully transcribed. An error occurred with a dictaphone in one interview, and 40 minutes did not record. This was realised immediately following the interview, and the researcher used the participant’s comprehensive timeline to help jog her memory and write detailed fieldnotes. The interview transcripts ranged from 5000 to 18,000 words, and included large sections of monologue from participants. Transcripts were imported into NVivo 11 for thematic analysis based upon themes that had been identified by the research questions and others which became apparent during data production (deductive), and those that became apparent during coding (inductive). Participant created visual materials were viewed alongside interview transcripts, but were largely treated as elicitation tools rather than data to be separately analysed. Melanie coded all data, and met with Aimee for regular analysis workshops. Codes relating to three pre-identified primary health behaviours - smoking during pregnancy, drinking alcohol during pregnancy and infant feeding – were then second coded throughout the entire data set by Aimee. Areas of differential coding were discussed and resolved.

Data from the codes were deductively mapped to the COM-B model [43] by one researcher Aimee. Each data extract from the codes relating to smoking, drinking alcohol or infant feeding, was assigned to one or more of the six COM-B domains within a Microsoft Word 2013 document. Following extraction, discussion occurred with the remaining members of the research team and a colleague familiar with the COM-B model to ensure appropriate coding [44]. Participants were not invited to provide feedback on the analysis, as our initial research design consultation with mothers suggested that this would be burdensome in a study which already required four hours of participants’ time. We focused on achieving a high level of ‘information power’ within our sample to adequately address the research questions, as opposed to the analysis aiming to achieve ‘saturation’, a contested concept within qualitative research, due to the pre-determined sample size [45]. Alongside the primary analysis reported in this paper, an in-depth sociological analysis was undertaken on data relating to smoking during pregnancy; this was reported separately [46].

## Results

A large body of data was collected. The life history interviews (phase 1) provided contextual information. Interviews two and three (phase 2) provided detailed accounts of health behaviours in pregnancy, including the three health behaviours reported here (smoking, drinking alcohol and infant feeding), alongside experience of sickness, being tired and diet. First, we present demographic details for the participants, alongside health behaviour in relation to maternal smoking and alcohol consumption and infant feeding experiences and intentions. Barriers and facilitators to meeting public health guidance in relation to the three core behaviours are then highlighted through the COM-B model.

### Demographics and self-reported health behaviours

Demographic details for participants are reported in Table 3; in order to preserve anonymity only two indirect identifiers are used in this table. The median age of participants was 29 (range 24–34). Of the ten participants, nine already had children; one previous child was the most common (*n* = 5). Only one participant was educated to degree level. The majority of participants were recruited during the first trimester of pregnancy. In self-reported health behaviours from their previous and current pregnancies were inconsistent (see Table 3). For example, Anna and Cat described complete abstinence from alcohol at some points of the interview, but later made reference to occasional or regular low level alcohol consumption. Anna commented: “Like I don’t go out, don’t drink…” but later stated: “We now have the odd drink in the house but we don’t go out.” Similarly, during her first interview (with Dunla, who was pregnant at the time) Catt said she had been easily able to quit smoking, but in her third interview (with Dawn) noted that she was regularly smoking a small number of cigarettes per day. These presentations of self, highlight the moral and identity work that pregnant women undertake to present as responsible, despite researchers attempts to present themselves as non-judgemental.

**Table 3:** Participant demographics and health behaviours

Pseudonym	Highest qualification	Parity (maternal age at birth (years))	Gestation (weeks) at recruitment	Smoking/e-cigarette use/smokefree environment during pregnancy	Alcohol use during pregnancy	Infant feeding experiences and intentions
Anna	NVQ 2	2 (23, 26)	8	Smoked on previous pregnancy, non-smoker during current pregnancy	Moderate* drinking during current pregnancy - “The odd one”	Attempted on first child, but moved to formula within first few days
						Plans to formula feed current baby
Becky	NVQ 2	1 (22)	18	Prior to previous pregnancy was smoking 15–20/day. Changed to e-cigarette and has remained an e-cigarette user throughout current pregnancy	Almost abstinent during current pregnancy - Not generally, but would have a small amount on special occasion (eg: her birthday).	Combination fed first child
						Plans to attempt to breastfeed
					Drank moderately during previous pregnancies	
Cat	NVQ 1	1 (23)	10	Smoker during current pregnancy – 1-2 per day	Moderate alcohol consumption during current pregnancy - One or two drinks	Did not attempt to breastfeed first child
						Conflicted about whether to try to initiate breastfeeding.
Donna	Degree	2 (28, 30)	20	Non-smoker; partner is a non-smoker; avoids smokey environments	Abstinent during current pregnancy; generally drinks a small amount	Attempted on first child, but moved to formula ‘quite quick’. Breastfed second child for 11 months.
						Plans to breastfeed current baby
Ellie	NVQ 2	1 (25)	10	Not discussed but home was not smokefree	Not discussed	Not fully discussed, but plans to buy a steriliser
Fiona	None	2 (17, 27)	9	Not discussed	Abstinent	Unclear how fed previous baby, but used a steriliser and bottle.
						Plans to formula feed current baby.
Gaby	GCSEs	3 (22, 24, 27)	6	Non-smoker	Abstinent – always abstinent	Tried to breastfeed three previous children (two quickly transitioned to formula; one breastfed for a month before transition to formula).
						Plans to formula feed current baby, but did state would try to initiate breastfeeding.
Hayley	A Levels	1 (29)	29	Non-smoker during current pregnancy – used to smoke	Abstinent during current pregnancy	Breastfed first baby for three months with one bottle of formula per day.
						Hopes to breastfeed current baby for at least two weeks.
					Almost abstinent during previous pregnancy would have a small amount on special occasion (eg: her birthday).	
Imogen	NVQ 2	1 (24)	8	non-smoker during current pregnancy	Abstinent during current pregnancy; Rarely drinks anyway	Not discussed, but had a traumatic birth and postnatal depression
Jess	GCSEs	0	11	Smoker – unsuccessful quit attempt in current pregnancy; trying to cut down	Abstinent during current pregnancy	Will attempt to initiate breastfeeding; plans to combination feed

*We defined 'moderate' drinking as one or two drinks on a regular or semi-regular basis

Most participants reported their planned infant feeding strategy confidently; either to choose to try to initiate breastfeeding (often with a caveat that it would be acceptable to them if this was not successful) or to immediately feed the baby with infant formula. By contrast, Cat was highly conflicted about whether to attempt to initiate breastfeeding, describing factors for and against attempting to initiate throughout the data production phase, highlighting several issues to be explored further in the COM-B analysis:

*“I have never liked, I don’t, obviously, disagree with it, I agree with it but I, it’s never, I’ve never, I couldn’t do it, it’s something that I don’t agree with myself doing it, I just can’t get the hang of it.”* (Cat)

### Barriers and facilitators to following public health guidance during pregnancy

Participants reported a range of barriers and facilitators in attempting to follow public health guidance, and occasionally they reported contradictions; with barriers and facilitators focused around the same COM-B domain. It was common for the issues that participants described to impact on multiple areas of the COM-B model. The findings are presented below in relation to remaining smoke free, alcohol consumption and infant feeding, and summarised in Table 4.

**Table 4:** Summary of themes arising during interviews corresponding to COM-B domains

Drivers of behaviour: barriers (−) and facilitators (+)	COM-B domain
Remaining smokefree during pregnancy	
Knowledge of potential harm (+), including from health professionals (+/−); high (+) or low (−) Carbon Monoxide test readings; belief that harm from smoking is real (+) or exaggerated (−)	Capability – psychological
	Opportunity - social
	Motivation - reflective
Pregnancy related nausea (+)	Capability - physical
Stress (−)	Capability - psychological
Stigma related to smoking during pregnancy and pressure to stop smoking leads to hidden smoking in the home and car (−); smoking in pregnancy normalised (−); strong family views that smoking is bad (+)	Opportunity – social
	Opportunity – environmental
	Motivation - reflective
Nicotine addiction (−); cravings to smoke (−); feeling that e-cigarettes were unable to deliver sufficient nicotine to reduce cravings (−); life long non-smoker (+); association between drinking alcohol and smoking leads to reduced cravings when not drinking alcohol (+)	Motivation- automatic
Remaining abstinent from alcohol during pregnancy	
Knowledge of guidelines (+/−) including from health professionals (+/−); belief that any alcohol is dangerous (+) or only heavy alcohol use is dangerous (−); belief that alcohol is dangerous reduces appeal (−)	Capability – psychological
	Opportunity - social
	Motivation - reflective
Pregnancy related nausea and tiredness reduce appeal (+); age and caring responsibilities for children make ‘hangovers’ unappealing (+)	Capability – physical
	Opportunity - social
	Motivation - reflective
Alcohol consumption in public is highly stigmatised (+/−); partners and family members encourage alcohol consumption in the home (−);	Opportunity - social
	Opportunity – environmental
Socialising largely takes place in premises serving alcohol and few non-alcoholic options (−); feelings of isolation from social group when not drinking alcohol (−)	Opportunity - social
	Opportunity – environmental
Willpower sufficient to resist alcohol (+); alcohol associated with relaxation and pleasure, leading to strong desire to drink alcohol (−)	Motivation- automatic
	Opportunity - social
Breastfeed exclusively for six months	
Knowledge of guidelines (+/−) including from health professionals (+/−); belief that breastmilk is superior to formula (+/−); belief that exclusive breastfeeding is important (+/−); belief that breastfeeding beyond the early days and weeks is important (+/−)	Capability – psychological
	Opportunity - social
	Motivation - reflective
Breastfeeding (+) or formula feeding (−) positioned as the convenient or ‘normal’ choice by the mother, and those around her; Formula feeding culture (−)	Capability – psychological
	Capability – physical
	Opportunity – social
	Motivation - reflective
Pressure to breastfeed from health professionals, family, friends and strangers in the antenatal and early post-natal period (−)	Capability – psychological
	Opportunity - social
Understanding of how to physically breastfeed (including latch and positioning) (+/−); physical challenges, including soreness, latch and tongue-tie (−); recovering from traumatic birth or C-section (−); support to overcome physical challenges from health professionals or others (+/−); hospitals have adequate/inadequate resources to support the initiation of breastfeeding (+/−)	Capability – psychological
	Capability – physical
	Opportunity – social
	Opportunity - environmental
Confidence in ability to breastfeed (+/−); inability to see how much milk baby is taking (−); belief in adequacy of milk supply (+/−); exposure to breastfeeding role models (+) or formula feeding role models (−); experience of formula feeding babies (−)	Capability – psychological
	Capability – physical
	Opportunity - social
Belief that it is OK to breastfeed in public (+); Confidence to breastfeed in public (+/−); belief in ability to ‘discreetly’ breastfeed in public (+/−); knowledge of ‘safe’ places to breastfeed in public (+); confidence-building support from health professionals or others (+)	Capability – psychological
	Capability – physical
	Opportunity – social
	Opportunity - environmental
Partners and family encourage bottle feeding to ‘help’ with care (−) – expressing breastmilk for bottle feeding takes additional time and maternal energy (−); mothers desire/ability to reject formula feeding (+/−); belief breastfeeding takes extra time (−) and lack of support for household chores and caring responsibilities (−)	Opportunity – social
	Motivation – reflective
	Capability – psychological
Breasts identified as sexual (−); Desire to stop breastfeeding to ‘get my body back’ (−) and quickly return to non-maternal activities/self identity (−)	Opportunity – social
	Motivation - reflective

#### Remaining smoke free during pregnancy

Guidance within the UK suggests that women should remain smoke free during pregnancy. The majority of the participants did not explicitly report that they had been told that the guidance was not to smoke, but this appeared to be because it was already a shared understanding between health professionals and pregnant women. Of the participants, two were current smokers, one was currently using an e-cigarette and three (including Becky who currently used an e-cigarette) had smoked during previous pregnancies. Of the remaining five participants, three reported they were non-smokers and smoking was not discussed with the final two participants (see Table 3). Both psychological and physical capability (see Table 1 for definitions of the COM-B model terms) was generally a facilitator to remaining abstinent, with knowledge of the potential harm to the foetus reported, and nausea and sickness a barrier to continual smoking:

*“I was smoking I can’t remember if it was 15 or 20 a day and then I fell pregnant with [daughter], and that was the whole scare factor again. But I did agree with what (the health professionals) were saying so I switched to (an e-cigarette) when I was pregnant with [daughter] and I’ve been on them for two and half years at least”* (Becky, e-cig user, ex-smoker)

*“But again that’s the sickness thing at the beginning (of pregnancy) and I wasn’t you know (smoking) comes hand in hand for me with drinking, or used to anyway…”* (Hayley, abstinent, ex-smoker)

For some participants who smoked prior to pregnancy, stress reduced psychological capability to abstain from smoking:

*“with (my most recent pregnancy)…towards the end, I had court with (my eldest daughter’s) father so seeing as I couldn’t drink, ok I did smoke five in a row because I was a bit stressed…”* (Anna, abstinent; smoker during previous pregnancy)

There was little social opportunity for pregnant women to smoke. Participants who had smoked during pregnancy described how they would “never” smoke in public, and reported the judgements they had experienced from strangers when smoking or using an e-cigarette:

*“I smoke my e-cig and some people…might look at me and like judge”* (Becky, e-cig user, ex-smoker)

Alongside this, the majority of pregnant smokers highlighted pressure to abstain from members of their immediate social network, such as partners. This disapproval, however, resulted in the home (and for Jess, her car) becoming a safe place in which to smoke:

*“When I was pregnant with [youngest daughter] and [eldest daughter] I did smoke with them but it was in my own house, I never walked around out and about with one, it’s not the best look.”* (Anna, abstinent; smoker during previous pregnancy)

*“My (car is my) bubble, I can cry, I can smoke, I can have a McDonalds in the car you know I can listen to music, I can do everything in the car.” (Jess, smoker)*

Those who had not smoked during a pregnancy reported a strong reflective motivation to not do so which affected their desire. By contrast, among women who smoked, the automatic motivation of addiction was prominent in discourses:

“*Even when I am not pregnant I don’t drink, I hate smoking, absolutely hate it. So yeah it’s never affected me because…I don’t miss it you know?”* (Gaby, never smoked)

“*No I smoked, I smoked and then I quit and then I, when I found out I did quit but then I started smoking again when I was pregnant and then I went onto those e-cig fags and then I stopped on that but now I am pregnant again I’ve started having a few fags again it’s like I’ve got a craving for smoke or something, it’s really weird”* (Cat, smoker)

#### Remaining abstinent from alcohol during pregnancy

Guidance within the UK suggested that women should remain abstinent from alcohol during pregnancy, although this guidance was introduced around four months prior to this research beginning, and previous guidance advised women could drink a small amount of alcohol each week. Of the participants, six reported they were abstinent from alcohol during their current pregnancy (one of whom had experience of low levels of drinking alcohol in a previous pregnancy), three reported that they currently drank alcohol and alcohol was not discussed with one participant.

Unlike smoking during pregnancy, where psychological capability was enhanced through a high level of knowledge of guidelines, participants reported that they did not always know that complete abstinence was recommended, with one participant being confused about the change in guidance:

*“…the alcohol thing changes all the time doesn’t it? Like I think with (previous pregnancy) it was a no-no and I think now you can have one, they don’t recommend it obviously…”* (Hayley, abstinent, drank alcohol during previous pregnancy)

Among women who were abstinent, knowledge of the potential negative impacts for babies in addition to the guideline were reported, and this impacted on reflective motivation:

*“I have seen like things where babies have had problems because their mothers have been drinking alcohol and it’s like it’s not fair on the baby and it’s how it makes me feel, when you have a drink you feel, sometimes you feel like whoa and I don’t want to put a poor innocent baby through that really.”* (Fiona, abstinent)

As with smoking during pregnancy, nausea or extreme tiredness resulted in some participants losing their physical capability to drink alcohol. Social opportunities for drinking alcohol were focused in two areas. First, three partners and family members encouraged alcohol consumption, which some participants resisted, but others did not:

*“I am sure, you can like (husband) has said oh I am sure it will be fine, just half a glass of wine.”* (Donna, abstinent)

*“I did have one by the end of [baby son] you know I think it was around Christmas time I had a glass because [partner]s mum and my mum was like oh we had one with you, you’re this far gone.”* (Hayley, abstinent, drank alcohol during previous pregnancy)

Second, participants described the centrality of alcohol to social occasions in their lives and how pregnancy disrupted this, either through removing their intention to drink alcohol, or by their consumption of alcohol being subjected to surveillance:

*“I miss the social aspect of that obviously you can’t drink when you’re pregnant, you can’t do a lot of things when you’re pregnant (…) I miss going out because I didn’t drink a lot before I was pregnant but now it’s just like you know you can’t and….”* (Jess, abstinent)

*“…in the end if my partner said anything I kind of just did the opposite just to you know prove a point, not, I wouldn’t go to the length that I would think that my baby is being harmed but just to make that point of you know that it’s nothing to do with you, this is my baby and my pregnancy. Because of Christmas we had I think it was champagne or wine or something and they were pouring it and my dad said: ‘Oh no, (Becky) can’t have any’, and I said: ‘Yes I can!’ [laughs]. I poured my share and made the point of drinking it all because it’s my baby and my pregnancy yeah. It irritates me so much when people say things like that because it’s not, and it’s men most of the time as well.”* (Becky, drinks alcohol occasionally)

This surveillance combined with a lack of alternatives to drinking alcohol resulted in participants feeling excluded from the night time economy. Accordingly, for those who did drink alcohol during pregnancy environmental opportunities were limited, and alcohol consumption was usually restricted to their home or the homes of friends.

Motivation to remain abstinent from alcohol was largely concentrated around reflective motivations. Among the abstinent participants, reflective motivation focused on the potential for drinking alcohol to harm the baby:

*“You know it can’t be that good (for the baby)”* (Hayley, abstinent, drank alcohol during previous pregnancy)

By contrast, participants who had experience of drinking alcohol in pregnancy reported an alternative belief about harms to the baby, which focused only on 'heavy' use as dangerous for babies:

*“I’ve had one or two drinks but some people they don’t mind drinking quite a lot when they’re pregnant but I don’t agree with that, I don’t mind having one or two, that can’t hurt you at all but I wouldn’t, I wouldn’t go over the limit do you know what I mean? I don’t agree with that but yeah I will have, I don’t do it often like…”* Cat, drinks alcohol regularly)

Alongside acknowledgements of harm, the desire to consume alcohol was not always reduced, and in some participants this required will power:

*“Oh I’d love one. (When we go on the hen night) It will kill me watching my mother with a bottle of wine, I’ll be there with my glass of coke [laughs]”.* (Anna, drinks alcohol occasionally)

#### Exclusive breastfeeding for six months

Overall, participants knew that breastfeeding was the recommended food for babies, but they did not generally know why this was, or the full extent of the guidelines to exclusively breastfeed for the first six months and to continue until the infant was at least two years old. Hayley reported the most detailed knowledge, but her knowledge of duration was incorrect:

*“Because of the goodness that can go through and strengthen the immune system (in the first 2 weeks) isn’t it? I can’t remember what they call it now… Especially when you get to six months and you don’t have any nutrients from breast milk so technically you should introduce a bottle by then…”* (Hayley, breastfed for three months previously; hopes to breastfeed)

Many participants who had previously been unsuccessful in breastfeeding their infants for as long as they had hoped reported reduced confidence (psychological capability), including in their physical ability to be able to breastfeed, and to breastfeed in public:

*“(I’m going to) bottle feed, yeah. I haven’t got the confidence to breastfeed. I am unsure really (if I’d like to try if I had the confidence), I lack confidence in that sort of thing so I would need confidence to do it but if I had the confidence I probably would give it a go.”* (Fiona, attempted to breastfeed but early exclusive formula feeding previously; plans to formula feed)

*“I’m not one of them people anyway even like when you’re out and about and that, I’m just a shy person like that, I couldn’t imagine myself doing it outside, I haven’t got the guts to do it.”* (Cat, formula fed previously; unsure whether to attempt to breastfeed)

Physical capability was highlighted as a major barrier to breastfeeding babies, including lack of understanding of how to breastfeed, tongue ties and traumatic births:

*“For the first two and half weeks you get, your boobs are so sore and you’re kind of just practising how to do it and they’re biting the wrong bits and sucking the wrong bit and then you get like bad blisters like all the way round and it’s so painful.*” (Becky, combination fed previously; hopes to breastfeed)

However, those who had breastfed beyond the early weeks noted that breastfeeding became the physically easy option:

*“you’d be amazed what you can kind of get done [laughs] when you’re feeding a baby. Whereas actually when you’re feeding a baby with a bottle, the kind of, it’s quite difficult you can’t really do much and I found that I was quite tied to a sofa”* (Donna, formula fed first child, exclusively breastfed second child, plans to breastfeed)

In relation to social opportunities, social norms could be a barrier and facilitator to breastfeeding, depending on whether they were focused on formula- or breast-feeding. For one participant who had previously formula fed, positive exposure to breastfeeding was a factor in her considering breastfeeding her forthcoming baby:

*“my brother’s girlfriend now with my niece, she is breastfeeding and she said it’s you know it’s so easy, you get such a closer bond as well so I am really thinking about it this time around.”* (Cat, formula fed previously; unsure whether to attempt to breastfeed)

Whilst social norms generally appeared relatively unobtrusive in participants’ accounts of initiating breastfeeding, when participants described infant feeding in public, it was clear that social opportunity played a major roll:

***“****everyone has got an opinion on (breastfeeding in public) and it’s quite, quite interesting listening to people and they’re like: ‘I’ll do it anywhere’. I’m not that sort of person, I wouldn’t just do it anywhere, I’d feed them you know I don’t know if we were in a café or if we were in a restaurant or we’d gone to I don’t know in a park on a bench somewhere. So yeah I think I’d still be mindful of other people because I understand that some people don’t want to, they don’t want to see it or they think it’s not right, it should be something that’s done at home…”* (Donna, formula fed first child, exclusively breastfed second child, plans to breastfeed)

Environmental opportunities were almost always highlighted as a barrier to breastfeeding, with inadequate support to learn how to physically breastfeed, and health service promotion of formula feeding:

*“And this one I am just thinking I am just going to do straight to the bottle because I was in hospital for an extra two nights trying to breastfeed [baby son] and not one of them picked up on the tongue tie…”* (Gaby, formula fed two previous children, breastfed third child for one month; plans to formula feed)

Alongside this, ‘help’ with feeding the baby was offered by partners and other family members:

*“I know (my husband) has got this system in place about feeding, how he will have the graveyard shift and I’ll have the rest of the day so I need to, in fact if it’s going to be breast obviously I’ve got to think about expressing and stuff like that. Like I said because he is going away (for work for a few months) you know the plan is at the moment him going away, I don’t have to worry about which it is.”* (Jess, first child, plans to attempt to breastfeed)

Participants reported mostly reflective motivations in their choice of infant feeding method. These focused on breastfeeding as “worth it” (Becky), or statements about the comparability of formula and breast milk:

*“I was bottle fed and there is nothing wrong with me so that’s what is in my head, I don’t think it’s wrong either way*.” (Hayley, breastfed for three months previously; hopes to breastfeed)

Alongside this, a major reflective motivation for stopping breastfeeding before the 24 month guideline was to “(get) my body back” (Donna; Gaby):

*“…although sometimes now, (my daughter) is still tugging at my top and I’m like, (daughter), no, get off (laughs). I was like no, it got to the point I thought I need my body back now, this is it now, you can have your milk, you can have a bottle.”* (Donna, formula fed first child, exclusively breastfed second child, plans to breastfeed)

### Absent voices

It is interesting to note that Imogen did not appear in the mapping of the data to the COM-B model, and this is because the health behaviour that mattered most to Imogen was maintaining a healthy weight and diet throughout her pregnancy, which was described at length during interviews. It may be that Imogen’s assertion that she does not drink or smoke made the space for this concern about weight and diet, that was much less present in the other interviewee’s accounts.

## Discussion

Among the ten participants from deprived areas involved in this research, none met the public health guidelines to be abstinent from alcohol [4] and tobacco [3] during pregnancy and to breastfeed their babies to 24 months and beyond [7, 8]. Overall, our findings mirror much of the previous research, often undertaken with more affluent women, as will be explored in more detail below. Knowledge about remaining abstinent from smoking was generally high, but lower in relation to remaining abstinent from alcohol and even lower in relation to breastfeeding beyond six months. Research into knowledge of alcohol guidelines in Australia has been inconsistent, with some studies highlighting mixed-messages and confusion [47] whilst others report high levels of knowledge, but lack of compliance with guidelines [6]. Previous research on knowledge of breastfeeding guidance from the UK Infant Feeding Survey reports that there is a strong social-class dimension to knowledge of the benefits of breastfeeding [48].

Furthermore, confidence to be able to breastfeed their babies, especially in public or in front of other people, was low in the majority of participants, further affecting psychological capability. Physical capability was generally high in relation to abstaining from alcohol and cigarettes, although it was a significant barrier to breastfeeding babies, with a range of factors relating to pain, latch and tongue ties. Confidence and physical capability to breastfeed – particularly when in a public setting - have been widely reported as barriers to breastfeeding in women from deprived [63] and more affluent backgrounds [49]. However, poverty presents additional barriers to physical and psychological capability [50], and should not be underestimated in the design of new interventions.

In relation to social opportunity, both smoking in public and drinking alcohol in public was viewed as unacceptable. Whilst on the face of it, this may seem as though it is a positive factor, women reported feeling stigmatised, judged and isolated. This finding mirrors previously recognised stigma against smokers in general [51], female smokers more specifically [52] and pregnant smokers as those breaching public health and feminine ideals [53]. Alcohol use generally is less stigmatised than smoking in the UK, and drinking small amounts during pregnancy is viewed as acceptable, whilst heavy drinking is demonised [54]. That said, concerns have been raised that interventions at the point of purchase may further stigmatise pregnant women, whilst having little effect on incidence of harm to babies [55]. We found that lack of social opportunity did not stop participants from smoking and drinking alcohol, as environmental opportunities in the shape of homes and other private spaces provided a safe place to engage in these behaviours.

One area of differences was that partners attempted to dissuade smoking during pregnancy, but facilitated and sometimes encouraged alcohol use during pregnancy. Research from the Netherlands also highlighted partners, the majority of whom had a university degree, encouraged light drinking in their pregnant partners [56]. Interestingly, the major social opportunity barrier that women reported in relation to breastfeeding was the need to breastfeed in public, which they feared would attract comments, looks and judgement which, again made them feel self-conscious and affected their confidence to breastfeed their babies at all. This has been widely reported in research with mothers, both in deprived [63] and affluent [49] areas of the UK.

Alongside this, environmental opportunities to negate physical capability challenges associated with breastfeeding were often reported to be inadequate. Midwives were often described as providing pressure in the antenatal period, but a lack of meaningful support in the immediate postnatal period, as has been reported in a repeat interview study with a mixture of women from deprived and affluent backgrounds in the UK [57]. Moreover, partners often offered help in the form of formula feeding babies, undermining exclusive breastfeeding, and in contrast to another study, which recruited male partners of women who breastfed exclusively for six months. These fathers used a range of other strategies to support infant feeding [58].

The final factor, motivation, was involved in all three behaviours. In relation to smoking, those who smoked stressed their automatic motivation through addiction and in some cases the beneficial effect that pregnancy-induced-nausea had on their ability to quit, as has been reported among indigenous women, a population where one in two women smoke [59]. By contrast, those who were abstinent highlighted their reflective motivation in the form of strong views that smoking was, and sometimes smokers were, bad, resonating with previous accounts of class-based othering [51].

In contrast, motivation to remain abstinent from alcohol was largely related to perceived ill effects for the baby, and among those who drank alcohol, a distinction was made between ‘safe’ and harmful levels of alcohol which was broadly in line with the previous UK guidance. This may be due to the relatively short period of time between the introduction of the new guidance and the data production period. Other explanations include a lack of discussion from midwives if women are perceived not to drink alcohol [60] or miscommunication [61].

Motivation in relation to breastfeeding was described less, although those with low confidence in their ability to breastfeed repeated their doubts in their ability to successfully nourish their babies, which is frequently reported among women who transition to formula feeding earlier than they had planned [48]. Alongside this, women who breastfed their babies reported getting to a point (from 1 to 11 months) where they wanted to stop breastfeeding to “get my body back”, articulating both the hyper-sexualisation of the breast in Western societies [62], and the greater burden of caring for infants which can fall to breastfeeding mothers if partners are not supportive [58].

In terms of further intervention development, we have highlighted partners, the public and health service staff as potential intervention participants and/or delivery partners to help facilitate these three behaviours. However, as the above comparison of the three behaviours in relation to the COM-B framework show, a range of approaches is necessary to facilitate a change in maternal behaviour. Accordingly, multi-behaviour interventions delivered through maternity services may not be appropriate for women during pregnancy [13]. Moreover, social and environmental opportunities should be considered, in relation to all three behaviours.

In relation to smoking and alcohol use in pregnancy in particular, we need to move away from an approach of individualised blaming [9], and consider how we can engage social networks in positive and supportive behaviours. In relation to breastfeeding, barriers to breastfeeding in public have been previously highlighted [63]**.** This is despite the Equality Act 2010 providing protection for women to breastfeed in public space. It is clear that some members of the public find viewing breastfeeding disgusting or distasteful [64], and interventions are urgently needed to normalise public breastfeeding, to provide a safe space for women who do wish to breastfeed.

### Strengths and limitations

This study aimed to gain a detailed understanding of health behaviours during pregnancy from the subjective accounts of pregnant women living in deprived areas of the UK and claiming means tested benefits. Consequently we worked with a small sample of ten women, who were interviewed three times, creating a nuanced qualitative data set in excess of 200,000 words. That said, our sample was not diverse in terms of ethnicity, and it is likely that the experiences of low income Black and minority ethnic women, framed by an additional lens of race and racism, would vary from our findings [65]. Furthermore, nine of the ten interviewees were already mothers, and their experiences may vary to women who are primigravida. Accordingly, a small sample of white women from a narrowly defined geographical area limits opportunities for generalisation. Additionally, we did not collect data on all three health behaviours from every participant and no details were requested in relation to whether pregnancies were low- or high-risk or whether obstetric diseases had occurred, although none were disclosed. A further limitation of our approach was a lack of participant validation of analysis.

Nevertheless, the research aims did not align with the quantitative sampling frame required to engender generalisability, and the positioning of participants as experts, necessarily produced data that emphasised participants, rather than researchers, salient areas of focus. Despite the lack of opportunity for participant validation of analysis, we undertook further stakeholder consultation with mothers and health professionals, which provided both a confirmation of the key themes reported, and additional insights into the next steps for this research.

## Conclusions

This paper highlighted a range of barriers to remaining abstinent from smoking and alcohol during pregnancy, and for breastfeeding among women from deprived areas of the UK. Mapping to the COM-B model illustrated the variation in barriers to achieving each behaviour. It is therefore unsurprising that multi-behaviour interventions often fail to achieve their multiple aims. Overall, the core influences in our three target behaviours appeared to be knowledge, confidence, partner support, and expert support to overcome physical challenges associated with addiction (smoking) and learning new techniques (breastfeeding). Alongside this, smoking, drinking alcohol (or even being present in places serving alcohol), and breastfeeding led to public condemnation of women, which may position them as failed maternal subjects. The need to retain bodily autonomy was also a barrier to breastfeeding.

Within the COM-B model, existing behaviours affect all three elements (Capability, Opportunity and Motivation); this should be considered within intervention design [24]. Importantly, this study has highlighted that when designing new interventions targeting women from deprived populations and our three target behaviours, it is important to co-produce the intervention with them in order to recognise the stigma and challenges to a good maternal-identity inherent within contemporary UK society. This involvement of women should not be a tokenistic exercise but one which is carefully designed to enable women to communicate their own subjective experiences and understandings of pregnancy and motherhood. In gaining an insight into the meaning making of women it is hoped that moves can be made to adopt interventions which are theoretically robust from the bottom up [66] which can potentially improve the health and wellbeing of mothers and their children.

## Additional file

Additional file 1: COREQ (COnsolidated criteria for REporting Qualitative research) Checklist (PDF 486 kb)

## Abbreviation

COM-B model: Capability, Opportunity, Motivation-Behaviour model
